# Advancing thoracic medicine through the Qatar International Thoracic Conference Abstracts

**DOI:** 10.5339/qmj.2024.qitc.1

**Published:** 2024-04-16

**Authors:** Merlin Thomas, Mousa Hussein, Irfan Ul Haq, Shakeel Ahmed, Tasleem Raza, Mansoor Hameed, Mushtaq Ahmed, Ibrahim Rashid, Muhanad Bilal, Mona Al Langawi, Hisham Abdul Sattar

**Affiliations:** 1 Department of Chest, Hamad Medical Corporation, Doha, Qatar Email: Mthomas27@hamad.qa; 2 Weill Cornell Medicine, Doha, Qatar; 3 College of Medicine, QU Health, Qatar University, Doha, Qatar

**Keywords:** Qatar International Thoracic Conference, conference abstracts

From the Scientific Planning and abstract committee of the Qatar International Thoracic Conference 2024, we are pleased to announce a significant step forward in the dissemination of research in the field of thoracic medicine. Reflecting our commitment to advancing medical science and enhancing patient care, the conference abstracts are now published under the DOI QITC, making them accessible to a global audience of healthcare professionals, researchers, and academicians.

The decision to publish the conference abstracts under DOI QITC was motivated by our desire to ensure the broadest possible access to these valuable insights. By making the abstracts readily available, we aim to foster a more informed and interconnected global medical community, where knowledge is shared freely, and collaboration is encouraged.

The abstracts cover a wide range of case reports and topics in thoracic medicine. Each abstract is a condensed but comprehensive overview of the interesting case reports and research offering readers insights into the study’s objectives, methodology, results, and potential implications for clinical practice.

Publishing these abstracts is more than just a procedural step; it is a reflection of our commitment to excellence in medical research and education. It is an opportunity to celebrate the hard work and dedication of the researchers, clinicians, and healthcare professionals who contribute to the advancement of thoracic medicine. By providing a platform for these findings, we hope to inspire further research and collaboration, ultimately leading to better patient outcomes and advancements in medical science.

In closing, the abstract committee of the Qatar International Thoracic Conference extends its gratitude to all contributors, reviewers, and attendees who made the conference a success. We are proud to share the published abstracts with the medical community and look forward to the continued advancement of thoracic medicine.

*For access to the published abstracts, please visit the* Qatar Medical Journal website.

## Conflict of Interest

None for all Authors.

